# Agreement between self-reported psychoactive substance use and urine toxicology results for adults with opioid use disorder admitted to hospital

**DOI:** 10.1080/24734306.2019.1700339

**Published:** 2019-12-09

**Authors:** Jessica L. Moreno, Matthew S. Duprey, Bryan D. Hayes, Kirsten Brooks, Sabrina Khalil, Sarah E. Wakeman, Russell J. Roberts, Jared S. Jacobson, John W. Devlin

**Affiliations:** a Department of Quality Management and Patient Safety, Beaumont Health, Southfield, MI, USA; b School of Pharmacy, Northeastern University, Boston, MA, USA; c Substance Use Disorders Initiative, Department of Medicine, Massachusetts General Hospital, Boston, MA, USA; d Department of Pharmacy, Massachusetts General Hospital, Boston, MA; e Department of Emergency Medicine, Harvard Medical School, Boston, MA, USA; f Department of Medicine, Harvard Medical School, Boston, MA, USA; g Department of Health Sciences, Northeastern University, Boston, MA, USA

**Keywords:** Drug screening, opioid related disorder, substance abuse detection, medication reconciliation

## Abstract

Hospitals often perform urine drug screens (UDS) upon inpatient admission to confirm self-reported psychoactive substance use for patients with opioid use disorder (OUD). We sought to evaluate the agreement between UDS and patient self-report for psychoactive substances detected with UDS for adults with OUD admitted to hospital. For 11 substance categories, we evaluated agreement between the UDS and the documented history over a 5-year period for consecutive adults admitted to one academic center with a history of OUD. Among the 153 patients, overall agreement across the 1683 different history/UDS pairs (i.e. either history+/UDS + or history−/UDS−) was high (81.3%) but varied (from lowest to highest) by substance [opiates (56.9%), benzodiazepines (66.0%), 6-acetylmorphine (67.3%), cocaine (81.0%), cannabinoids (81.0%), methadone (83.7%), buprenorphine (85.0%), amphetamine (94.8%), barbiturates (95.4%), and phencyclidine (98.7%)]. History+/UDS− pair mismatches were most frequent for 6-acetylmorphine (32.7%), methadone (14.3%) and oxycodone (12.4%); history−/UDS + pair mismatches were most frequent for opiates (43.1%), benzodiazepines (24.8%) and cannabinoids (18.3%). The change in agreement over time of self-reported heroin use may reflect an increasing number of patients unknowingly using illicit fentanyl products. Among hospitalized patients with OUD, agreement between reported psychoactive substance use history and UDS results is strong with the exception of opiates, heroin, and benzodiazepines.

## Introduction

More than 2 million Americans currently have opioid use disorder (OUD) and deaths from opioid overdose have quintupled since 1999 [1]. The nation’s overdose crisis is characterized in three waves; the first beginning with increased opioid analgesic prescribing in the 1990s, the second from a rapid rise in heroin-related overdose deaths starting in 2010, and a third from an exponential rise in overdose deaths associated with illicit fentanyl products beginning in 2013 [2]. Alarmingly, illicit fentanyl products are increasingly identified in products sold as heroin [3]. As more people are affected by this crisis, many eventually interact with the healthcare system, often for acute care needs [4, 5].

Hospitalizations among patients with OUD more than doubled over the past decade [6]. Additional substance use and psychiatric comorbidities are common in individuals with OUD who often self-administer both prescribed and non-prescribed psychoactive substances [7]. Patients with OUD require a thorough review of medication and substance use at the time of admission. This helps ensure individuals receive adequate OUD treatment while hospitalized, risk factor(s) for a withdrawal syndrome(s) are recognized, other substance-related medical issues are identified, and the scope of current substance use is accurately characterized.

While some suggest urine toxicology screening should require informed consent, urine drug screens (UDS) often occur at the time of admission to confirm self-report or when patients cannot communicate their use [8-10]. For hospitalized patients with OUD, agreement between self-reported use and the admission UDS remains unknown. We therefore sought to measure the agreement between self-reported psychoactive substance use and UDS results among hospitalized patients with OUD.

## Methods

We undertook an IRB-approved, retrospective, secondary analysis of a cohort of patients admitted for OUD [11]. We used the Partners Research Patient Data Registry (RPDR) to electronically identify consecutive adults admitted to one academic medical center in Boston, MA between October 1, 2011 and September 30, 2016 with either an ICD-9-CM or ICD-10 diagnostic code or problem list item suggestive of OUD (e.g. ICD-9-CM: 304 opioid dependence, 965.09 poisoning by other opiates and related narcotics; ICD-10: F11.10 opioid abuse-uncomplicated, F11.23 opioid dependence with withdrawal, etc.) [12, 13].

We included patients with a UDS conducted within 24 h of hospital admission that reported on all of the following substances: 6-acetylmorphine (6-AM; heroin metabolite), amphetamines, barbiturates, benzodiazepines, buprenorphine, cocaine, methadone, opiates, oxycodone, phencyclidine, and cannabinoids. We excluded patients with a UDS completed ≥ 24 h after admission to avoid potential detection of in-hospital psychoactive medication administration. We identified self-reported substance use through documented medication and substance use histories and excluded patients if both were not documented.

Over the 5-year study period, the hospital utilized the Cobas C501 analyzer (Roche Diagnostics; Indianapolis, IN) for all urine drug screen immunoassays. Table 1 summarizes target substances and test characteristics as specified by each assay manufacturer [14-24]. In this analysis, “opiates” refers to the UDS immunoassay panel of multiple opioids. For the purposes of this analysis, we did not seek confirmatory test results to establish agreement with patient self-report. Rather, we determined agreement between patient self-report and UDS using presumptive positive results of the screening immunoassays.

Trained data extractors collected data from the electronic medical record system [from October 1, 2011 to March 31, 2016 using the Longitudinal Medical Record (LMR) system (an in-house system developed at Partners Healthcare System) and thereafter using Partners eCare [developed in conjunction with Epic (Verona, WI)]. We used Research Electronic Data Capture (REDCap), a secure, web-based application for validated data entry, transmission, and storage to manage all extracted data.

We analyzed data on an individual level rather than in aggregate to avoid ecological fallacy. We cross-referenced each UDS result with the patient’s self-reported history. We recorded agreement for each UDS result-history pair when the agent was present in the UDS and the patient reported taking it or when the agent was not present in the UDS and the patient denied taking it. We calculated descriptive and comparative statistics using SAS software version 9.4 for MS Windows (SAS, Cary, NC).

## Results

**Figure 1.**
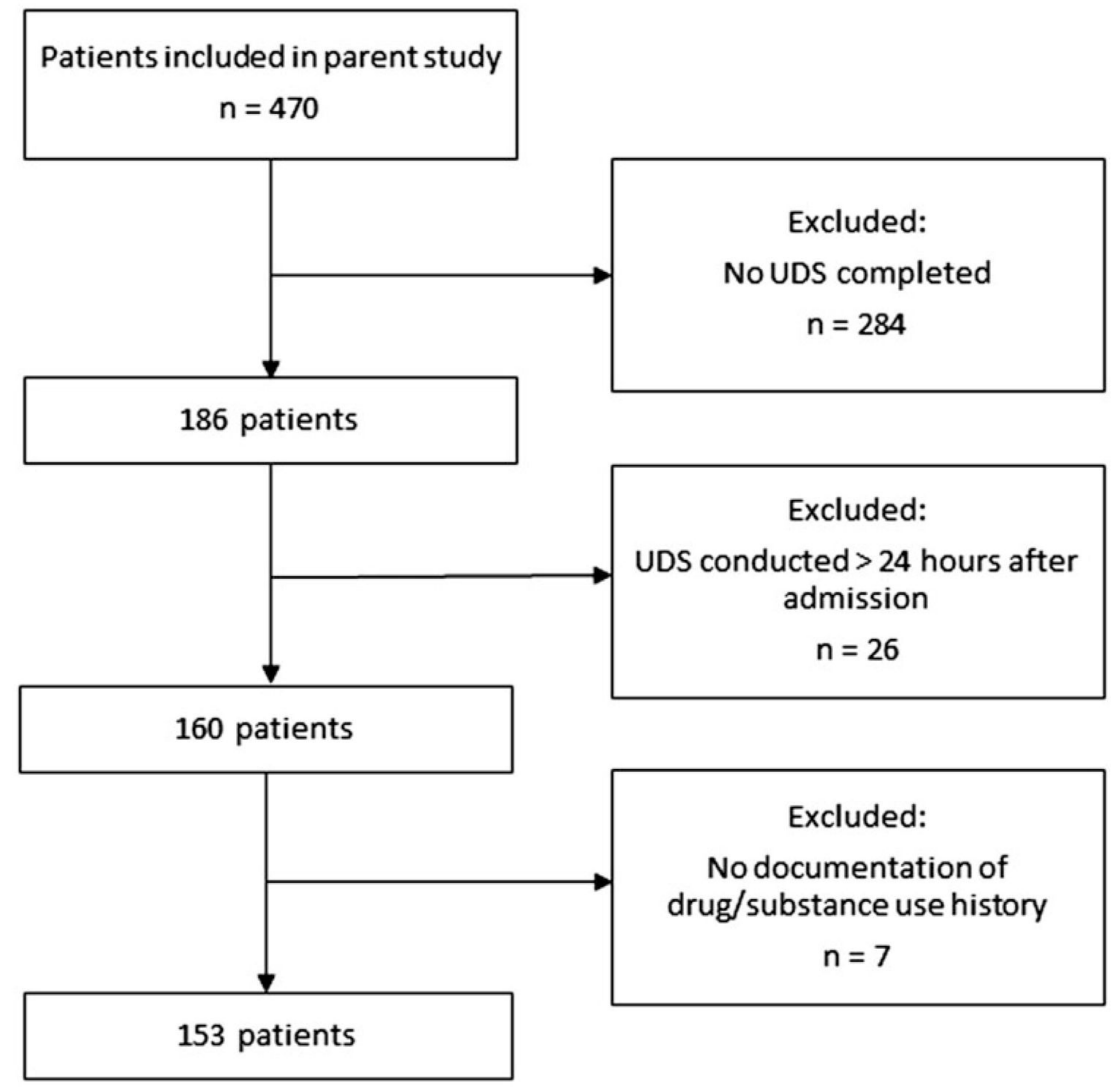
Flowchart of patient inclusion.

Among the 470 patients in the parent cohort, 160 (34.0%) had a UDS within 24 h of hospital admission, 26 (5.5%) had a UDS completed over 24 h after admission, and 284 (60.4%) never had a UDS completed. Among the 160 patients with a UDS within 24 h of admission, 7 (4.4%) did not have a medication/substance use history documented in their medical records and thus we included 153 patients in the final analysis (Figure 1). Table 2 presents the patient characteristics.

The 153 patients represented a total of 1683 different UDS/history pairs. Table 3 illustrates the frequencies of agreement and disagreement between UDS results and histories. Overall paired agreement was high (1369/1683 = 81.3%). Agreement was highest for phencyclidine (151/153 = 98.7%), barbiturates (146/153 = 95.4%), and amphetamines (145/153 = 94.8%) and lowest for 6-AM (103/153 = 67.3%), opiates (87/153 = 56.9%), and benzodiazepines (101/153 = 66.0%).

Disagreements (positive UDS/negative history and negative UDS/positive history) were common (Table 3). There were three potential false-positive UDS results (amphetamine + due to trazodone; benzodiazepine + due to sertraline; and methadone + due to quetiapine) [25]. Of note, 36 of the 50 UDS-negative 6-AM cases occurred after 2012, aligning with the timing of increasing prevalence of illicitly manufactured fentanyl product distribution [26].

## Discussion

Previous studies of agreement between UDS results and self-reported substance use history focused on patients receiving care in outpatient or emergency department (ED) settings. In the present study of hospitalized patients, agreement between patient self-report and UDS results was common for all substances (>80%), with the lowest level of agreement for opiates, benzodiazepines, and 6-AM (57–67%). The level of overall agreement between self-report and UDS was comparable to a prospective analysis of patients receiving psychiatric consultation in the ED [8]. These investigators reported overall agreement between self-report and UDS of 85.3%. Disagreement was most common in cases when patients reported cannabis or alcohol use but these substances were not detected *via* UDS. In a prospective, cross-sectional study, Rashidian *et al*. [10] evaluated the sensitivity of self-report and UDS to detect opioid use in healthy individuals and hospitalized patients. Sensitivity of self-report was comparably high for hospitalized patients (77.5%) and occurrence of positive UDS results when patients denied use, was similarly rare (7.9%). The frequent agreement between self-report and UDS across multiple studies demonstrates that UDS does not usually appear to provide more information than what a patient is already willing to acknowledge.

The higher rates of positive UDS for opiates and benzodiazepines in our study (when patients did not report use of these substances) may indicate non-prescribed use of these agents in individuals with OUD. This may occur when patients do not feel comfortable disclosing their use out of fear of stigmatizing or punitive approaches taken by clinicians treating them. Health care workers in various treatment settings are identified as a common source of stigma towards patients with OUD [27]. It is possible patients would be more open to disclosing their use if clinicians were trained on compassionate approaches when treating this patient population.

Patients frequently reported heroin use, but had negative results for opiate and 6-AM screens. One explanation may be the short time window for detection of 6-AM [28]. Alternatively, patients may have thought they used heroin and thus reported doing so, when instead, they unknowingly used something else. This scenario is increasingly likely since 2013, when many regions of the United States, including New England, started seeing a dramatic rise in distribution of illicitly manufactured fentanyl (IMF) [29]. In our study, 72% of the UDS that were negative for 6-AM when a patient reported using heroin occurred after 2012, coinciding with the rising rates of IMF distribution. Fentanyl was not included as an agent analyzed in the UDS panels utilized at our center, therefore we were unable to confirm its presence in this subset of samples.

However, 83% of patients presenting to a community ED in Baltimore, MD for treatment of OUD, overdose, or withdrawal tested positive for fentanyl [30]. Another recent investigation of non-hospitalized volunteers with self-reported use of heroin or IMF from Dayton, OH compared self-reported use of these substances to results of UDS [31]. These researchers found that individuals who reported use of heroin, but denied use of IMF, frequently had UDS positive for IMF products, suggesting these individuals were unaware of the contents of their supplies. The addition of fentanyl to standard UDS testing may be warranted, as suggested in a study showing over 96% of patients presenting to a New England ED following suspected heroin overdose tested positive for nonpharmaceutical fentanyl [32, 33]. False-positive interference is also a known issue with UDS immunoassays [34].

Our analysis has limitations. First, due to the retrospective nature of our study, we were unable to characterize the quality of the medication and substance use histories conducted, thus their reliability remains uncertain. Second, patients with OUD admitted to our center might represent a different demographic from those at other centers and thus our results may limit external validity. Additionally, only one-third of patients from the initial cohort met inclusion criteria for this study. Third, our use of results from screening immunoassays, rather than confirmatory testing, introduces potential for inaccuracies. Future prospective analyses could utilize confirmatory testing to compare with self-report to minimize false-positive rates. Finally, it is possible that a positive UDS with negative history could occur in a patient who received a therapeutic dose of medication before the urine collection occurred. Due to the retrospective nature of our study and limitations of electronic medical record documentation in the earlier years of our data collection window, we could not confirm that each specimen was collected prior to administration of any of the screened substances.

Given the inherent limitations of UDS along with evidence that patients with OUD are mostly accurate in their reporting of substance use, the utility of UDS in this patient population may have a narrower scope than is often employed at healthcare centers. One option is to rely on patient reports of substance use and reserve UDS for patients who are unable to communicate or are unsure of what they may have used. With this arrangement, clinicians might be better able to build rapport by including patients as members of the care team and demonstrating their trust towards them (rather than skepticism). In cases when patients can communicate, it is reasonable to obtain informed consent prior to conducting any toxicology testing.

## Conclusions

Agreement between patient self-report and UDS among hospitalized patients with OUD was high. Frequencies of agreement were lower for opiates, 6-acetylmorphine, and benzodiazepines than for other substance tested. The increasing frequency of reported heroin use with negative UDS in later years likely reflects the transition to illicit fentanyl use. In cases when UDS is considered warranted, adding a screen for fentanyl may increase agreement.

## Figures and Tables

**Table 1.** Summary of urine drug screen immunoassay specifications.

Drug	Assay Manufacturer	Immunoassay	Cut-off Value	Tests For (Approximate % Cross-Reactivity)^a^	False Positives^b^
6-acetylmorphine	Microgenics Corporation (Fremont, CA)	CEDIA^®^ Heroin Metabolite Assay [14]	10ng/mL	6-acetylmorphine	Structurally unrelated compounds were tested with the CEDIA Heroin Metabolite (6-AM) Assay and gave a negative response when tested.
Amphetamines	Roche Diagnostics (Indianapolis, IN)	Amphetamines II [15] AM3Q2:ACN 814 AM5Q2:ACN 815 AM1Q2:ACN 816 AM3S2:ACN 817 AM5S2:ACN 818 AM1S2:ACN 819 AM5QC:ACN 787	300ng/mL 500ng/mL 1000ng/mL	*d,l*-3,4-Methylenedioxymethamphetamine (255) *d,l*-3,4-Methylenedioxyamphetamine (127) *d*-Methamphetamine (102) *d*-Amphetamine (101) *d,l*-N-Methyl-1-(3,4-methylenedioxyphenyl)-2-butanamine hydrochloride (84) *d,l*-3,4-Methylenedioxyethylamphetamine (75) *d,l*-3,4-Methylenedioxyphenyl-2-butanamine hydrochloride (37) *l*-Methamphetamine (11)	The cross-reactivity for LSD was tested at a concentration of 2500 ng/mL. The results obtained were 1.89 %, 1.76 %, and 1.43 %, for the 300 ng/mL, 500 ng/mL, and 1000 ng/mL assay cutoffs respectively. The cross-reactivity for Δ9-THC-9-carboxylic acid was tested at a concentration of 10,000 ng/mL. The results obtained were 0.56 %, 0.49 %, and 0.44 %, for the 300 ng/mL, 500 ng/mL, and 1000 ng/mL assay cutoffs respectively.
Barbiturates	Roche Diagnostics (Indianapolis, IN)	Barbiturates Plus [16] BA2QP:ACN 572 BA2SP:ACN 573	200 ng/mL	Cyclopentobarbital (101) Aprobarbital (93) Butalbital (71) Allobarbital (71) Butabarbital (37) Pentobarbital (36) Amobarbital (29) Phenobarbital (22) *p*-Hydroxyphenobarbital (19) Barbital (11)	None of the tested compounds gave values in the assay that were greater than 0.012% cross-reactivity.
Benzodiazepines	Roche Diagnostics (Indianapolis, IN)	Benzodiazepines Plus [17] BZ1QP: ACN 611 BZ2QP: ACN 612 BZ3QP: ACN 613 BZ1SP: ACN 615 BZ2SP: ACN 616 BZ3SP: ACN 617	100 ng/mL 200 ng/mL 300 ng/mL	Nordiazepam Demoxepam (99) Estazolam (94) Diazepam (93) Alprazolam (91) α-Hydroxyalprazolam (88) 4-Hydroxyalprazolam (81) α-Hydroxyalprazolam glucuronide (54) Triazolam (85) α-Hydroxytriazolam (82) 4-Hydroxytriazolam (80) Clorazepate (85) Clobazam (84) Bromazepam (83) Nitrazepam (81) 7-Aminonitrazepam (84) Temazepam (78) Oxazepam (77) Flunitrazepam (71) 7-Aminoflunitrazepam (94) Desmethylflunitrazepam (73) 3-Hydroxyflunitrazepam (56) Pinazepam (69) Clonazepam (65) 7-Aminoclonazepam (70) Lormetazepam (65) Midazolam (65) α-Hydroxymidazolam (75) Chlordiazepoxide (63) Desmethylchlordiazepoxide (58) Prazepam (59) Lorazepam (59) Flurazepam (57) Hydroxyethylflurazepam (88) Desalkylflurazepam (88) Didesethylflurazepam (73) Halazepam (57) Medazepam (51) Desmethylmedazepam (33)	None of the tested compounds gave values in the assay that were greater than 0.031% cross-reactivity for the 100 ng/mL cutoff, 0.05% cross-reactivity for the 200 ng/mL cutoff, and 0.022% cross-reactivity for the 300 ng/mL cutoff.
Buprenorphine	Microgenics Corporation (Fremont, CA)	CEDIA^®^ Buprenorphine Assay [18]	5 ng/mL	Buprenorphine Buprenorphine-3-β-D Glucuronide (98)	All pharmacologic compounds evaluated were <0.015% cross-reactive in the CEDIA^®^ Buprenorphine Assay.
Cannabinoids	Roche Diagnostics (Indianapolis, IN)	Cannabinoids II [19] TH2Q2: ACN 441 TH5Q2: ACN 442 TH1Q2: ACN 443 TH2S2: ACN 444 TH5S2: ACN 445 TH1S2: ACN 446	20 ng/mL 50 ng/mL 100 ng/mL	11-nor-Δ^9^ THC-9-carboxylic acid 9-carboxy-11-nor-Δ^8^ THC (71.9) 9-carboxy-11-nor-Δ^9^ THC glucuronide (44.1) 8-β-11-dihydroxy-Δ^9^ THC (33.9) 8-α-hydroxy-Δ^9^ THC (13.0) 11-hydroxy-Δ^9^ THC (11.6)	For the 20 ng/mL cutoff, the cross-reactivity for Niflumic Acid, at a concentration of 1250 ng/mL, is 2%. For the 50 ng/mL cutoff, the cross-reactivity for Niflumic Acid, at a concentration of 4750 ng/mL, is 1%. For the 100 ng/mL cutoff, the cross-reactivity for Niflumic Acid, at a concentration of 10,897 ng/mL, is 1%.
Cocaine	Roche Diagnostics (Indianapolis, IN)	Cocaine II [20] CO1Q1: ACN 189 CO3Q2: ACN 267 CO1S2: ACN 268 CO3S2: ACN 477	150 ng/mL 300 ng/mL	Benzoylecgonine	None of the tested compounds gave values in the assay that were greater than 0.05% cross-reactivity.
Methadone	Roche Diagnostics (Indianapolis, IN)	Methadone II [21] MD3Q0: ACN 447 MD3S0: ACN 448	300 ng/mL	Methadone	Caution should be taken when interpreting results of patient samples containing structurally related compounds having greater than 0.5% cross-reactivity with 300ng/mL assay cutoff. These include Hydroxymethadone, Cyamemazine, Methotrimeprazine (Levomepromazine), and Chlorpromazine The cross-reactivity for Disopyramide at a concentration of 1 mg/mL was tested with the Methadone II assay. The result obtained was <0.01%. Specimens from Seroquel (quetiapine fumarate) users have screened positive for methadone. The cross-reactivity for Tramadol, at a concentration of 102,465 ng/mL, is 0.3%. The cross-reactivity for Ofloxacin, at a concentration of 220,000 ng/mL, is 0.1%.
Opiates	Roche Diagnostics (Indianapolis, IN)	Opiates II [22] OP3Q2: ACN 497 OP2Q2: ACN 495 OP3S2: ACN 498 OP2S2: ACN 496	300 ng/mL 2000 ng/mL	Codeine (134) Ethyl morphine (101) Diacetylmorphine (82) 6-Acetylmorphine (78) Dihydrocodeine (59) Morphine-3-glucuronide (54) Hydrocodone (28) Thebaine (25) Hydromorphone (21)	The cross-reactivity for Rifampin was tested with the Opiates II assay. The results obtained were 16.8% and 6.9% for the 300 ng/mL and 2000 ng/mL cutoffs, respectively.
Oxycodone	Microgenics Corporation (Fremont, CA)	DRI^®^ Oxycodone Assay [23]	100 ng/mL 300 ng/mL	Oxycodone (100) Oxymorphone (103)	All of the pharmacologic compounds evaluated, including a number of the opiate compounds, exhibited no cross-reactivity at the concentrations tested.
Phencyclidine	Roche Diagnostics (Indianapolis, IN)	Phencyclidine Plus [24] PC2QP:ACN 518 PC2SP:ACN 519	25 ng/mL	Phencyclidine	The cross-reactivity for Amitriptyline, Desipramine, and Imipramine were tested at a concentration of 100,000 ng/mL with the Phencyclidine Plus assay. The results obtained were 0.031%, 0.022%, and 0.037%, respectively.

a Approximate percent cross-reactivity specified if package insert included it. If value was less than 10%, we did not include the compound.

b The assay manufacturers did not test for cross-reactivity with all known potential false positive triggers [34].

**Table 2.** Study cohort patient demographics.

Characteristic	*n* = 153
Age (years)^a^	41 ±12
Male gender	96 (63%)
White race	136 (89%)
Insured via Medicaid	129 (84%)
Self-reported heroin use (vs. prescription opioid)	127 (83%)
Psychiatric comorbidities	
Non-opioid substance use disorder	78 (51%)
Major depressive disorder	70 (46%)
Anxiety disorder	61 (40%)
Bipolar affective disorder I or II	28 (18%)
PTSD	20 (13%)
Other	30 (20%)
Reason for admission	
Infection	41 (27%)
Neurological disorder	23 (15%)
Substance use	19 (12%)
Gastrointestinal/hepatic/renal	17 (11%)
Other	53 (35%)

a Mean ± standard deviation. All other results presented at N (%).

PTSD = post-traumatic stress disorder.

**Table 3.** Paired comparison between history and urine drug screen for each substance across the study cohort (*n* = 153).

Substance	Agreement N (%)			Disagreement N (%)		
	HX + UDS +	HX − UDS −	Matched Pairs	HX+UDS −	HX − UDS +	Non-Matched Pairs
6-acetylmorphine	2 (1.3)	101 (66.0)	103 (67.3)	50 (32.7)	0 (0)	50 (32.7)
Amphetamine	9 (5.9)	136 (88.9)	154 (94.8)	6 (3.9)	2 (1.3)	8 (5.2)
Barbiturate	0 (0)	146 (95.4)	146 (95.4)	0 (0)	7 (4.6)	7 (4.6)
Benzodiazepine	41 (26.8)	60 (39.2)	101 (66.0)	14 (9.1)	38 (24.8)	52 (34.0)
Buprenorphine	20 (13.1)	110 (71.9)	130 (85.0)	18 (11.8)	5 (3.2)	23 (15.0)
Cannabinoids	13 (8.4)	111 (72.5)	124 (81.0)	1 (0.6)	28 (18.3)	29 (19.0)
Cocaine	27 (17.6)	97 (63.3)	124 (81.0)	6 (3.9)	23 (15.0)	30 (19.0)
Methadone	17 (11.1)	111 (72.5)	128 (83.7)	22 (14.3)	3 (1.9)	25 (16.3)
Opiates	11 (7.2)	76 (49.7)	87 (56.9)	0 (0)	66 (43.1)	66 (43.1)
Oxycodone	9 (5.9)	121 (79.1)	130 (85.0)	19 (12.4)	4 (2.6)	23 (15.0)
Phencyclidine	0 (0)	151 (98.7)	151 (98.7)	0 (0)	2 (1.3)	2 (1.3)

Abbreviations: HX = history, UDS = urine drug screen, ‘+’ = present, ‘−‘ = not.
